# Beyond the Phase Segregation: Probing the Irreversible Phase Reconstruction of Mixed‐Halide Perovskites

**DOI:** 10.1002/advs.202103948

**Published:** 2021-12-19

**Authors:** Zhe Li, Xin Zheng, Xuan Xiao, Yongkang An, Yanbo Wang, Qingyi Huang, Xiong Li, Rongrong Cheacharoen, Qinyou An, Yaoguang Rong, Ti Wang, Hongxing Xu

**Affiliations:** ^1^ School of Physics and Technology and Key Laboratory of Artificial Micro‐ and Nanostructures of Ministry of Education Wuhan University Wuhan 430072 China; ^2^ Wuhan National Laboratory for Optoelectronics Huazhong University of Science and Technology Wuhan 430074 China; ^3^ State Key Laboratory of Advanced Technology for Materials Synthesis and Processing Wuhan University of Technology Wuhan Hubei 430070 China; ^4^ State Key Laboratory of Metal Matrix Composites Shanghai Jiao Tong University Shanghai 200240 China; ^5^ Metallurgy and Materials Science Research Institute Chulalongkorn University Bangkok 10330 Thailand

**Keywords:** mixed‐halide perovskite, phase reconstruction, photoluminescence imaging, stability

## Abstract

Mixed‐halide perovskites can undergo a photoinduced phase segregation. Even though many reports have claimed that such a phase segregation process is reversible, what happens after phase segregation and its impact on the performance of perovskite‐based devices are still open questions. Here, the phase transformation of MAPb(I_1−_ *_x_* Br*_x_*)_3_ after phase segregation and probe an irreversible phase reconstruction of MAPbBr_3_ is investigated. The photoluminescence imaging microscopy technique is introduced to in situ record the whole process. It is proposed that the type‐I band alignment of segregated I‐rich and Br‐rich domains can enhance the emission of the I‐rich domains by suppressing the nonradiative recombination channels. At the same time, the charge injection from Br‐rich to I‐rich domains drives the expulsion of iodide from the lattice, and thus triggers the reconstruction of MAPbBr_3_. The work highlights the significance of ion movements in mixed‐halide perovskites and provides new perspectives to understand the property evolution.

## Graphical abstract

Photoluminescence imaging microscopy is employed to investigate the phase transformation of MAPb(I_1−_ *_x_* Br*_x_*)_3_ under continuous irradiation. Due to the charge injection from Br‐rich to I‐rich domains, an irreversible reconstruction of MAPbBr_3_ is induced. This work reveals the mechanisms of photo‐induced phase reconstruction, and deepens the understanding of the stability of mixed‐halide perovskites.

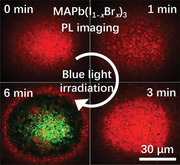

## 1 Introduction

Hybrid lead halide perovskites have emerged as an exciting class of highly efficient optoelectronic materials due to their unique properties, such as high absorption coefficient, long charge carrier diffusion length, and tunable bandgaps (*E*_g_), etc.^[^ ^1^, ^2^, ^3^, ^4^, ^5^ ^]^ Typical perovskites have a formula of APbX_3_, where A is an organic/inorganic monovalent cation (methylammonium, MA^+^, formamdinium, FA^+^, and cesium, Cs^+^), and X is a halide anion (Cl^−^, Br^−^, and I^−^).^[^ ^6^, ^7^, ^8^ ^]^ Since the first report of using MAPbX_3_ as the light‐harvesting material for photovoltaics by Miyasaka et al. in 2009,^[^ ^1^ ^]^ perovskite solar cells (PSCs) have rapidly developed in the next decade, achieving a world record power conversion efficiency (PCE) of 25.5%.^[^ ^9^, ^10^, ^11^, ^12^, ^13^ ^]^ Besides photovoltaics, perovskites have also demonstrated promising potential for the applications of light‐emitting devices, lasers, photodetectors, and high‐energy radiation detection.^[^ ^14^, ^15^, ^16^, ^17^ ^]^

While the research interest on perovskite‐based optoelectronics is initiated by MAPbI_3_, the state‐of‐the‐art devices usually employ binary/ternary cation and double‐halide perovskites (e.g., Cs_1−_ *_y_*_−_ *_z_* FA*_y_* MA*_z_* Pb(I_1−_ *_x_* Br*_x_*)_3_) as the light‐absorbing layer.^[^ ^18^, ^19^, ^20^, ^21^ ^]^ Tuning the compositions of A site has been proved effective for improving the moisture/heat stability of perovskites.^[^ ^22^, ^23^, ^24^ ^]^ For the X site, mixing I^−^ and Br^−^ anions permits direct *E*_g_ control between pure APbI_3_ (*E*_g_ ≈1.5 eV) and APbBr_3_ (*E*_g_ ≈2.4 eV),^[^ ^25^ ^]^ which has therefore spawned significant interest in developing wide‐bandgap perovskites for constructing multijunction tandem solar cells.^[^ ^26^, ^27^, ^28^ ^]^ Despite the high performance, the intrinsic instability of perovskites against moisture, heat, and illumination, etc., makes it challenging for PSCs to achieve operational lifetimes that meet the requirements for commercial applications.^[^ ^29^, ^30^ ^]^ Although the external influence can be effectively isolated by proper encapsulations,^[^ ^31^, ^32^ ^]^ the restrain of internal failure of materials and interfaces still relies on the understanding of the degradation kinetics and mechanism.

For mixed‐halide perovskites of APb(I_1−_ *_x_* Br*_x_*)_3_, notorious photoinduced phase segregation under continuous‐wave irradiation has been observed.^[^ ^33^ ^]^ The formation of a narrow bandgap I‐rich phase may cause a severe reduction of the device photovoltage.^[^ ^34^ ^]^ Such phase segregation is considered reversible by monitoring the photoluminescence (PL) emission, and a myriad of microscopic models have been developed to rationalize it, including defect‐driven^[^ ^35^, ^36^, ^37^ ^]^ and polaron‐induced lattice strain driven^[^ ^38^, ^39^, ^40^, ^41^ ^]^ segregation of halide ions. Particularly, Brabec et al. demonstrate that the photoinduced phase segregation selectively takes place at grain boundaries rather than within grain centers.^[^ ^42^, ^43^ ^]^ Then, Kamat et al. report that the hole accumulation induces iodide to move from the lattice toward grain boundaries in perovskite thin films.^[^ ^44^ ^]^ By selectively injecting holes into the mixed‐halide perovskite through electrochemical anodic bias, iodide is gradually expelled from the thin film leading to the reformation of MAPbBr_3_ domains.^[^ ^45^ ^]^ Thus, it is highly possible that the phase segregation under continuous‐wave irradiation accompanies reconstruction of crystallized perovskite domains, and may result in an irreversible transformation of perovskite thin films.

Here, we find that the previously reported reversible phase segregation will immediately happen under the blue light irradiation (mercury lamp, ≈460–490 nm, ≈13.0 W cm^−2^) forming I‐rich and Br‐rich domains. The I‐rich domains gradually collapse and the phase reconstruction of MAPbBr_3_ subsequently occur, which is recorded by in situ PL imaging microscopy technique. Particularly, it is demonstrated that the PL emission at 730 nm first enhances (photobrightening phenomenon) and then vanishes (phase reconstruction). The photobrightening is caused by the type‐I band alignment of highly segregated I‐rich and Br‐rich phases which can significantly suppress the nonradiative recombination in I‐rich domains. Meanwhile, the charge injection from Br‐rich to I‐rich domains induces the expulsion of iodide from the lattice and thus triggers the reconstruction of MAPbBr_3_. By constructing a stacked double‐layer perovskite thin films of MAPbI_3_ on the top of MAPbBr_3_, the efficient energy transfer is verified from MAPbBr_3_ to MAPbI_3_. With a blue excitation light, the thin film only shows emission at 760 nm, without any trace of MAPbBr_3_ in the spectra. Thus, the band alignment benefits the emission of the I‐rich domains on one hand and triggers the collapse of the I‐rich lattice on the other hand. Our work systematically monitors the structural and compositional evolution of mixed‐halide perovskites under continuous‐wave irradiation from phase segregation to reconstruction and provides essential insights into their influence on the long‐term stability of perovskite‐based electronics devices.

## 2 Results and Discussion

**Figure 1:**
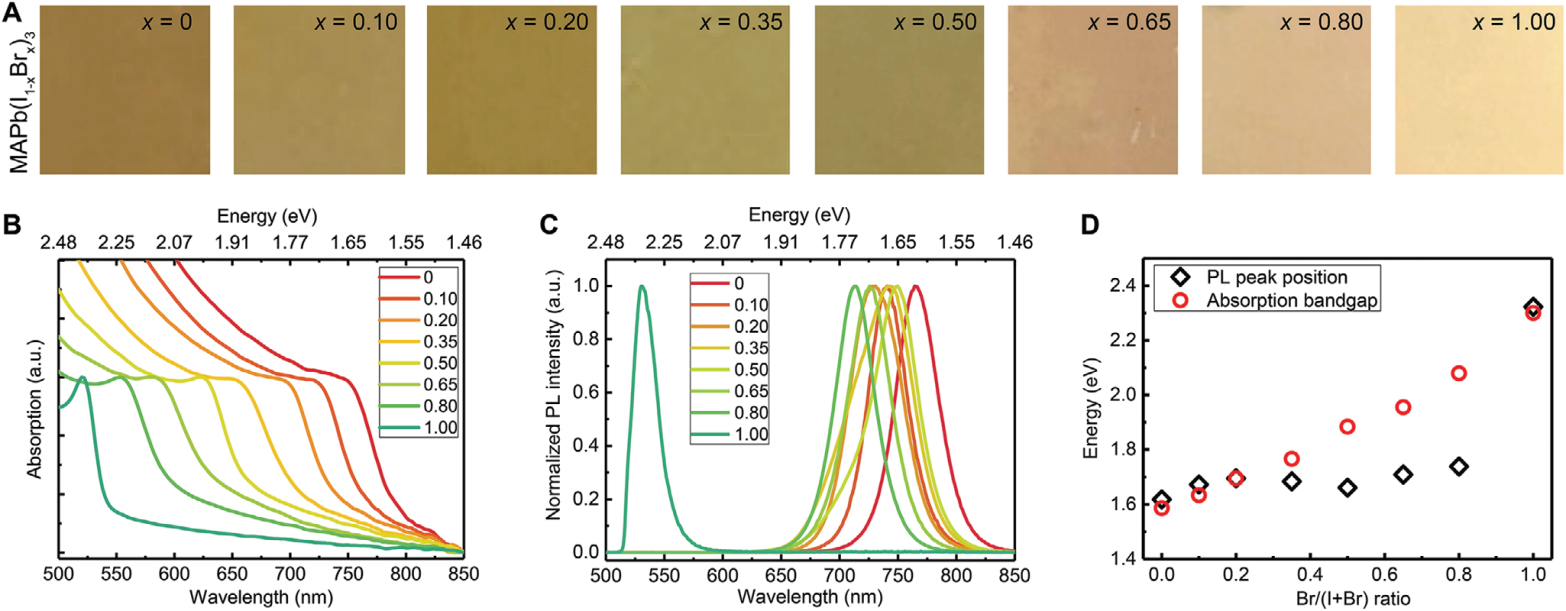
Optical characterization of mixed‐halide perovskite thin films. A) Digital images of MAPb(I_1‐_ *_x_* Br*_x_*)_3_ thin films on the cover glass. B) The UV–vis spectra of MAPb(I_1−_ *_x_* Br*_x_*)_3_ thin films on the cover glass. C) Steady‐state PL spectra of MAPb(I_1−_ *_x_* Br*_x_*)_3_ thin films on the cover glass. (The spectra are collected using a Halogen lamp with the excitation density of 1.04 W cm^−2^, and the integration time is 10 s for each spectrum). D) The comparison of PL peak positions and calculated absorption bandgaps of MAPb(I_1−_ *_x_* Br*_x_*)_3_.

Mixed‐halide perovskite thin films with the composition of MAPb(I_1−_ *_x_* Br*_x_*)_3_ (*x* = 0–1) were deposited on glass substrates by spin‐coating perovskite precursors. The experimental details are described in the Supporting Information. As the ratio of Br in the perovskites increases, the color of the films turns from brown to light yellow, as shown in **Figure** **1**A. To distinctly probe the phase reconstruction processes, the thicknesses of all the films are set to ≈80 nm. The absorption spectra of the perovskite films are characterized by UV–vis spectra (Figure 1B). Being consistent with the results in the literature,^[^ ^25^ ^]^ the onset of the spectra continuously shift from ≈808 nm (1.53 eV, pure MAPbI_3_) to ≈540 nm (2.30 eV, MAPbBr_3_) as the ratio of Br increases, indicating continuously tunable bandgaps by mixing Br ions. The optical bandgaps have been extracted from the absorption spectra which are shown in Figure 1D. An almost linear curve is obtained as the value of *x* increases.

However, the PL peak positions are not located correspondingly with the absorption spectra, as shown in Figure 1C. MAPbI_3_ and MAPbBr_3_ thin films present PL emission peaks at 1.61 and 2.34 eV, respectively, which are consistent with other works.^[^ ^33^, ^46^ ^]^ Nevertheless, for 0.2 < *x* < 0.8, all the PL peaks distribute between 1.65 and 1.77 eV. Particularly, MAPb(I_0.5_Br_0.5_)_3_ should have a distinct optical bandgap of 1.85 eV as indicated by the absorption spectra. Intriguingly, the PL peak of MAPb(I_0.5_Br_0.5_)_3_ locates at 1.70 eV. Even for *x* = 0.65 and 0.80, the PL peaks are located below 1.77 eV, which is far beyond expectations from the absorption spectra (Figure 1D). According to previous works, the shift of PL peaks is considered as the result of photoinduced phase segregation under continuous wave (CW) irradiation.^[^ ^33^ ^]^ In general, the irradiation causes the initial emission of MAPb(I_1−_ *_x_* Br*_x_*)_3_ (0.2 < *x* < 0.8) to redshift from the origin positions toward 1.70 eV which is the existence of a terminal *x* = 0.2 value. This observation is consistent with the work first reported by Hoke and his colleagues.^[^ ^33^ ^]^ This critical value is irrespective of the initial MAPb(I_1−_ *_x_* Br*_x_*)_3_ stoichiometry. Although the mechanism of this phenomenon is still under debate, a plausible scenario involves a cubic‐to‐tetragonal phase transition at *x* ≈0.2 in MAPb(I_1−_ *_x_* Br*_x_*)_3_. In addition, no light‐induced spectral shifts are observed below *x* = 0.2 which shows a similar trend with the absorption spectra.

It should be mentioned that all the PL spectra in Figure 1C are extracted after the phase segregation process. It is noted that the mixed‐halide perovskites (0.2 < *x* < 0.8) are apt to phase segregate and the rates of phase segregation processes are proportional to the intensity of the irradiation light. Under the excitation density of 1.04 W cm^−2^, the thin films have simultaneously segregated with the light on. A low irradiation light intensity and/or a short integration time are prerequisites to observe the initial PL spectra of MAPb(I_1−_ *_x_* Br*_x_*)_3_ (*x* > 0.2) (Figure S1, Supporting Information). Since this work concentrates on the reconstruction processes, the phase segregation processes are not discussed here in detail. For instance, the phase segregation of MAPb(I_0.5_Br_0.5_)_3_ is investigated with a 532 nm Continuous Wave (CW) laser with a power density of 680 mW cm^−2^ (Figure S2, Supporting Information). We note that even under this low‐intensity excitation, the initial PL peak of MAPb(I_0.5_Br_0.5_)_3_ is almost disappeared after 1 min of illumination. Moreover, we further lower down the excitation density one more order with our detection limit (68 mW cm^−2^) and the initial peak has shifted to 720 nm after 100 s. Therefore, we can confirm that it is quite easy and fast for phase segregation to take place in mixed‐halide perovskites, and the process is highly sensitive to illumination intensity and flux.

**Figure 2:**
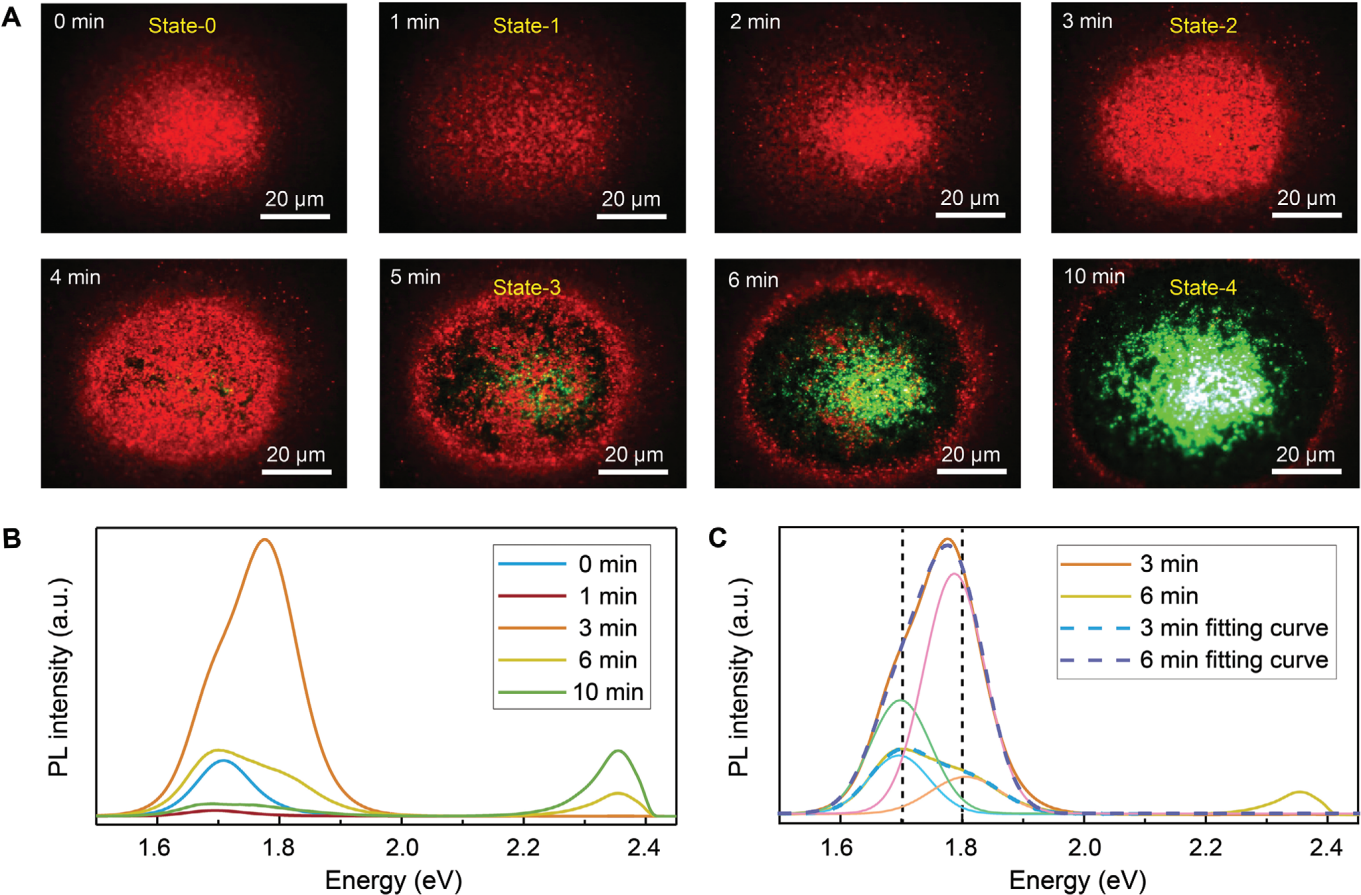
In situ observation of the reconstruction of mixed‐halide perovskite. A) Evolution of the PL imaging results of MAPb(I_0.5_Br_0.5_)_3_ thin films under continuous blue‐light irradiation (Mercury lamp, 13 W cm^−2^). B) The corresponding PL spectra at 0, 1, 3, 6, and 10 min. C) The fitting results of PL spectra for 3n and 6 min irradiation.

The PL measurements show that the photoinduced phase segregation in mixed‐halide perovskite thin films occurs almost simultaneously with the light on. Irrespective of the initial *x* value, the thin films exhibit a PL emission at around 1.70 eV. To in situ investigate the mixed‐halide perovskite thin film behaviors under light irradiation, the PL imaging microscopy technique has been utilized here to record this process (Figure S3, Supporting Information). Since the maximum intensity of solar spectra is at ≈460–490 nm, a condition close to reality is selected to model the effect of blue‐light on mixed‐halide perovskites. Here, the light source is a mercury lamp with an ≈460–490 nm bandpass filter, and the light source spectrum is presented in Figure S4 (Supporting Information). Here, we use the MAPb(I_0.5_Br_0.5_)_3_ thin film as a demonstration to in situ investigate the processes after phase segregation. **Figure** **2**A depicts the evolution of PL imaging results of MAPb(I_0.5_Br_0.5_)_3_ thin films taken at various irradiation times under continuous blue‐light irradiation. Meanwhile, the correlated PL spectra are recorded and illustrated in Figure 2B.

Noteworthy, there are different processes during the irradiation which we have denoted as five states. First, at the beginning (0 min), phase segregation has already occurred in the thin film with a homogenous red emission (State‐0). Only one PL emission peak at 1.70 eV can be observed. Second, the halide ions start to migrate through the thin film with a decreased PL intensity (State‐1). Third, the red PL peak intensity begins to increase after 2 more min irradiation and reaches the maximum at 3 min (State‐2). Fourth, the red emission decreases, and a green emission appears after 5 min irradiation corresponding to a new PL emission peak at 2.34 eV (State‐3). Fifth, the red emission at the center almost disappears and the whole irradiation area emits green light (State‐4). To distinctly illustrate the whole process, a video has been presented in the Supporting Information. Moreover, Figure 2B presents the PL spectra at different irradiation times corresponding to the PL images in Figure 2A. For State 0, only one PL peak at 1.70 eV is observed. However, for Sate‐2, the maximum PL peak position shift to 1.79 eV and has a shoulder on the high energy side. The PL spectra of 6 min have a similar feature as that of 3 min.

Since the phase segregation has occurred instantaneously after irradiation, what we observe here are the processes after phase segregation which can be simply considered as the decomposition and reconstruction processes. Intuitively, if the mixed‐halide perovskite thin films are continuing degraded under the blue‐light irradiation, the PL intensity should monotone decrease. However, two counter‐intuitive phenomena have been observed. First, the PL intensity abnormally increases at a certain time (State‐2) during the decomposition process. Second, instead of turning into a mess, the phase segregated thin films induce MAPbBr_3_ reconstruction (State‐4) under irradiation. It should be noticed that all the thin films with initial *x* values have remarkably similar processes. The whole processes of MAPb(I_0.35_Br_0.65_)_3_ thin film have also been shown in the Supporting Information which presents a similar direction as MAPb(I_0.5_Br_0.5_)_3_ (Figure S5, Supporting Information).

To investigate the origin of the PL emission at 3 min, the PL spectra are fitted by two Gaussian peaks, which are located at 1.70 and 1.80 eV, respectively (Figure 2C). The 1.70 eV position relates to *x* value of 0.2, while 1.80 eV is the PL emission position of MAPb(I_0.5_Br_0.5_)_3_. After 1 min irradiation, the PL emission decreases and has already split into two peaks indicating the change of the composite in the thin film. There are two possible reasons for the reappearance of the 1.80 eV PL peak. First, the MAPb(I_0.5_Br_0.5_)_3_ has formed by ion migration. Recent work has observed that halide ions diffuse in 2D halide perovskite vertical heterostructures with an interdiffusion rate of about 10^−19^ to 10^−20^ m^2^ s^−1^.^[^ ^47^ ^]^ Thus, the halide ions can migrate under light irradiation and form back to the initial composite after 1 min. Second, the decomposition induces inefficient energy transfer. The PL emission of the initial composite is caused by efficient energy transfer in the mixed‐halide thin film. The phase segregation process occurs instantaneously under blue‐light irradiation which forms I‐rich and Br‐rich regions. However, only the PL emission of I‐rich domains can be observed due to the efficient energy transfer process. After 1 min irradiation, the decomposition of the thin film impedes the energy transfer process in the thin film which induces the reappearance of initial MAPb(I_0.5_Br_0.5_)_3_ emission. Moreover, the decomposition also results in the decrease of the whole PL intensity during the first 1 min.

For the 3 min PL spectrum, both the PL intensities of MAPb(I_0.5_Br_0.5_)_3_ and MAPb(I_0.8_Br_0.2_)_3_ are stronger than the initial. Quitsch et al. find a photobrightening effect when the photon energy above a critical wavelength of ≈520 nm and a photodarkening effect below this wavelength. They attribute this phenomenon to the decomposition process for MAPbI_3_.^[^ ^48^ ^]^ It is noted that MAPb(I_1−_ *_x_* Br*_x_*)_3_ can decompose into MAI, PbI_2_, and PbBr_2_, and PbX_2_ can passivate defects and grain boundaries to suppress the nonradiative recombination.^[^ ^49^ ^]^ Thus, a possible mechanism is the decomposition components of PbI_2_ and PbBr_2_ passivate the defects and grain boundaries which induces an increase of PL intensities. However, the process (State‐2) observed here is reversible. If we stop the irradiation at State‐2, the thin film will go back to the initial state (Figure S6, Supporting Information). This indicates the decomposition has not started at this state. Instead, we deduce that the increased PL emission is due to the formation of type‐I band alignment between the highly segregated I‐rich and Br‐rich domains. This energy band alignment significantly suppresses the nonradiative recombination in I‐rich domains and grain boundaries, and correspondingly enhances the PL intensity. A similar idea has been proposed in perovskite solar cells. By inserting large bandgap 2D perovskites, the nonradiative recombination channels can be reduced at the 2D–3D junction.^[^ ^50^ ^]^

Notably, the evolution starts to turn irreversible after State‐2. After 3 min irradiation, the PL intensity begins to decrease again, and a new PL emission peak at 2.34 eV can be observed in the PL spectra. This process can be considered as the reconstruction process. Charge carriers in Br‐rich domains will transfer into I‐rich domains, which induce the expulsion of iodide. Particularly, the injected holes may oxidize the iodide ions, leading to the formation of iodine and/or triiodide, either in the bulk or at surfaces/grain boundaries. Noteworthy, molecular iodine is unstable in the thin film. If it forms in the bulk, I_2_ thus tends to migrate to the surface, where it can easily escape from the thin film. MAPb(I_1−_ *_x_* Br*_x_*)_3_ keeps decomposing and forms I_2_ which will leave the thin film. Meanwhile, the Br ions migrate to the Br‐rich region and reconstruct MAPbBr_3_. It is noted that the green light has first appeared at the edge of some black holes where there are no red PL emissions. Thus, we speculate the MAPbBr_3_ first reconstructs at the grain/domain boundaries (Figure 2A) and the reconstruction process must be caused by anionic migration.^[^ ^35^, ^51^ ^]^ Although the mechanism of anionic migration is still under debate, a possible reason is due to the ability of halides to migrate through halide defects in the perovskite structure. Grain/domain boundaries always have more defects than the center of a grain/domain. Thus, it is reasonable that the reconstruction process first occurs at grain/domain boundaries. At 10 min, the red emission at the irradiation area almost disappears indicating the complete decomposition of the thin film. Meanwhile, the green light becomes stronger demonstrating the reconstruction of MAPbBr_3_ in the whole area.

To study the impact of the irradiation power, we have performed experiments under various irradiation densities. Noteworthy, the reconstruction process can be observed with different irradiation densities (Figure S7, Supporting Information). When the irradiation density decreases, it takes a longer time for the whole reconstruction process. Interestingly, if the irradiation density lowers down one order, the required time of the reconstruction process increases 10 times. A possible explanation is that the reconstruction process depends on the irradiation flux (Figure S8, Supporting Information). This result can fully support our proposed mechanism of phase reconstruction. Higher irradiation density will generate more electrons and holes which results in more charges injecting into I‐rich domains. This increased injection will speed up the phase reconstruction process. Thus, the reconstruction rate is dependent on the number of excited electrons and holes and the whole reconstruction process is proportional to the irradiation density. Moreover, to exclude the impact from laser heating, the MAPb(I_0.5_Br_0.5_)_3_ samples have been heated up to 100 ℃ for 10 min in the atmosphere (Figure S9, Supporting Information). It shows the same PL emission as the initial state which does not have the green emission. Thus, the laser heating effect should not induce the phase reconstruction of MAPbBr_3_.

**Figure 3:**
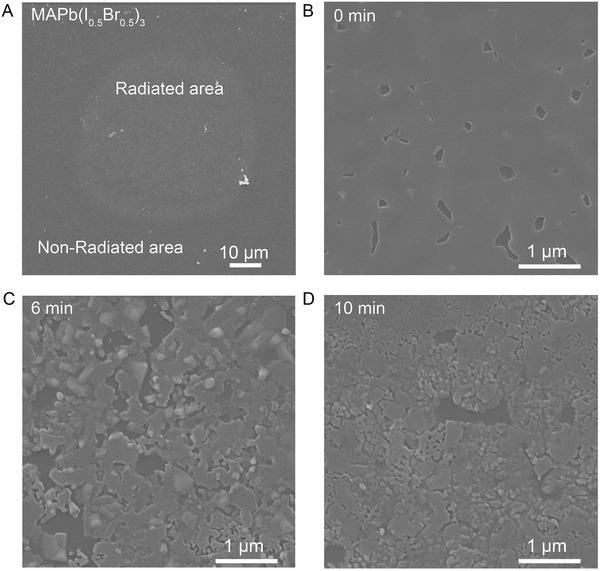
Morphology characterization. A) The surface SEM images of MAPb(I_0.5_Br_0.5_)_3_ thin films irradiated by blue excitation light with low magnifications. B−D) The surface SEM images of MAPb(I_0.5_Br_0.5_)_3_ thin films irradiated by blue excitation light after different time with high magnifications.

To investigate the morphology evolution of the mixed‐halide perovskite film (MAPb(I_0.5_Br_0.5_)_3_) along with phase reconstruction, we perform top‐down scanning electron microscopy (SEM) measurements. As shown in **Figure** **3**A, the radiated area is significantly distinguishable, showing a circle shape with a diameter of ≈50 µm which is consistent with the mercury lamp beam size. For the nonradiated area, it presents compact morphology with fragmentary polygonal voids (Figure 3B). After 3 min irradiation, the film shows a similar feature as the nonradiated area, which further confirms the reversible process until this state (Figure S10, Supporting Information). When the film is exposed to blue light for 6 min, the voids enlarge, and at the same time, the compact area begins to decompose into interconnected island‐shape domains (Figure 3C). When the film is further irradiated for 10 min, the size of the domains becomes smaller, for which the shape is similar to pristine PbI_2_ or PbBr_2_ (Figure 3D).

**Figure 4:**
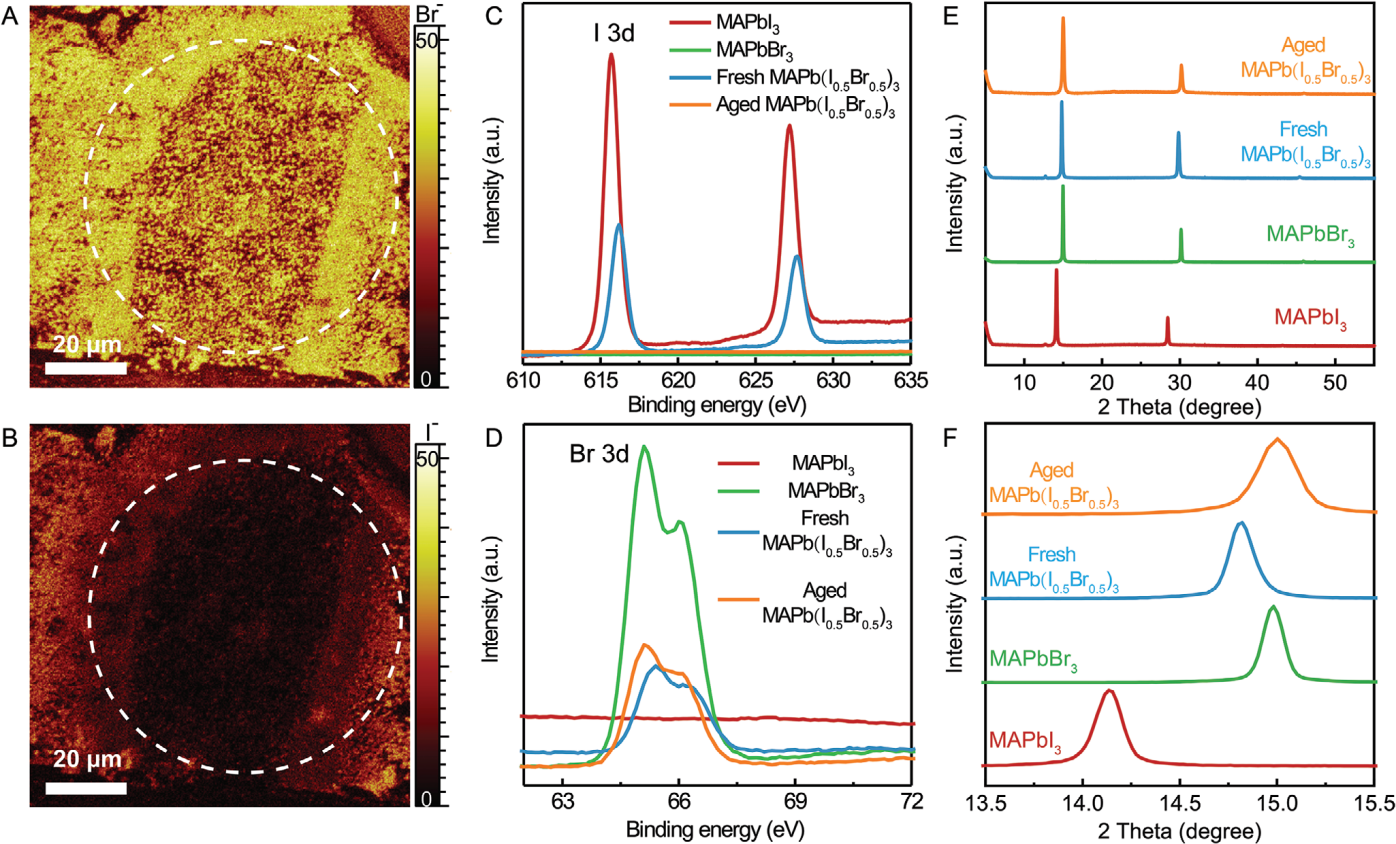
Element and structural characterization. A,B) TOF‐SIMS mapping signals of Br^−^ and I^−^ on the MAPb(I_0.5_Br_0.5_)_3_ thin film, and the irradiated area is marked with white dash line circles. C,D) The XPS spectra and E,F) XRD patterns of MAPbI_3_, MAPbBr_3_, fresh MAPb(I_0.5_Br_0.5_)_3_, and aged MAPb(I_0.5_Br_0.5_)_3_ thin films.

Considering the morphology evolution, we propose that the decomposition of MAPb(I_0.5_Br_0.5_)_3_ has occurred during the continuous blue‐light irradiation. To verify this issue, we first analyze the halide distribution around the irradiated area by time of flight secondary ion mass spectrometry (TOF‐SIMS), as shown in **Figure** **4**A,B. The microscopy and PL imaging results are also presented for comparisons (Figure S11, Supporting Information). After irradiation, the signals of Br^−^ ions show reconstructed domains/patterns at the center, although the profile does not overlap with that in the microscopy image. This could be due to the inhomogeneous intensity of the beam spot. On the contrary, the signals of I^−^ ions become quite weak after irradiation, and the profile matches with that of Br^−^ signals. This indicates the reconstruction of the Br‐rich phase/domains accompanies by the collapse of I‐rich phase/domains. According to the literature, the complexation between Pb^2+^ and Br^−^ is nearly 7 times greater than the complexation between Pb^2+^ and I^−^, thus making Br^−^ a dominant binding species in mixed‐halide perovskites.^[^ ^52^ ^]^

X‐ray photoelectron spectroscopy (XPS) measurements are further conducted. The core‐level spectra of I_3d_ and Br_3d_ for fresh and irradiated (aged) MAPb(I_0.5_Br_0.5_)_3_ films are compared, and the spectra of MAPbI_3_ and MAPbBr_3_ films are employed as the reference, as shown in Figure 4C,D. For the fresh MAPb(I_0.5_Br_0.5_)_3_ film, both I and Br can be detected. After 10 min irradiation, no I signal can be detected while the signals of Br stayed almost unchanged. Normally, the loss of I ions is considered as the result of the evaporation of volatile MAI. However, in this case, PbI_2_ still should give signals of I. As we mention the mechanism previously, the charge injection from Br‐rich into I‐rich domains will result in the oxidization of iodide ions. At the same time, the Pb^2+^ will be reduced to Pb^0^ (Figure S12, Supporting Information). Since XPS can only detect elements on the surface of the thin films, we also conducted X‐ray diffraction (XRD) measurements to extract structural information of the films. The XRD patterns of fresh MAPb(I_1−_ *_x_* Br*_x_*)_3_ films with different *x* values are collected and compared to confirm that bromine has completely incorporated in the crystal lattice (Figure S13, Supporting Information). For MAPb(I_0.5_Br0_.5_)_3_, as shown in Figure 4E,F, the fresh film exhibits a peak at 14.7°, which is between the characteristic peaks of MAPbI_3_ and MAPbBr_3_. After irradiation, the peak shifts to the position of MAPbBr_3,_ and no other peaks can be observed in the XRD results. This indicates that the thin film has completely phase reconstructed to MAPbBr_3_. Moreover, the full width at half maximum of the MAPbBr_3_ peak becomes wider than the initial one and directly deposited MAPbBr_3_ thin film which illustrates a lower crystalline quality. Notably, the aged MAPb(I_1.5_Br_1.5_)_3_ film does not involve the signals of crystalline PbI_2_. We have prepared pristine PbI_2_ thin films on glass substrates, and aged them under Mercury lamp irradiation for 30 min. According to XRD measurements (Figure S14, Supporting Information), the crystalline PbI_2_ has not significantly decomposed under blue light irradiation. Thus, the aged MAPb(I_1.5_Br_1.5_)_3_ film may contain PbI_2_ in an amorphous state.

**Figure 5:**
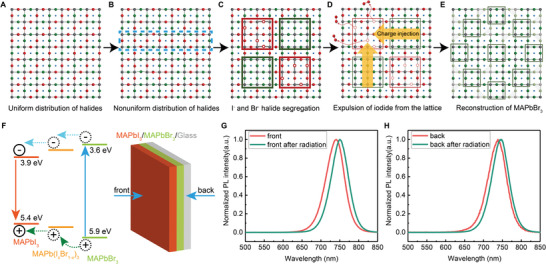
Mechanism of the reconstruction of MAPb(I_0.5_Br_0.5_)_3_ A–E) Scheme for the evolution from halide segregation of MAPb(I_0.5_Br_0.5_)_3_ to a reconstruction of MAPbBr_3_. F) The energy level diagram and energy transfer between MAPbI_3_, MAPb(I_0.2_Br_0.8_)_3_, and MAPbBr_3_. G,H) The PL spectra of bilayer perovskite MAPbI_3_/MAPbBr_3_ on cover glass measured from the front and back sides.

With the experiment results above, we schematically propose the reconstruction process in **Figure** **5**A–E. The uniform thin film has been prepared with the stoichiometric of MAPb(I_0.5_Br_0.5_)_3_ (Figure 5A). Under blue‐light irradiation, the halide ions migrate in the thin film, leading to inhomogeneous distribution (Figure 5B). Furthermore, the film segregates simultaneously into I‐rich and Br‐rich regions (Figure 5C). At this state, a maximum PL intensity can be observed at 1.70 eV. With the increasing time of blue‐light irradiation, the charge injection from Br‐rich domains starts to induce the decomposition of I‐rich domains by expelling the iodide ions out of the lattice (Figure 5D). The possible mechanism is sketched as follows

(1) $$\begin{array}{c} \begin{array}{c} n\mathrm{MAPb}{\left(I_{0.5}Br_{0.5}\right)}_{3}\overset{h\nu }{↔}m\mathrm{MAPb}{\left(I_{0.5+x}Br_{0.5-x}\right)}_{3}+m\mathrm{MAPb}{\left(I_{0.5-x}Br_{0.5+x}\right)}_{3}\\ \;\;+\left(n-2m\right)\mathrm{MAPb}{\left(I_{0.5}Br_{0.5}\right)}_{3}\;\;\;\;\mathrm{Phase}\;\mathrm{segregation} \end{array} \end{array}$$

(2) $$2I^{-}+2h^{+}\to I_{2}\;\;\;\mathrm{Iodine}\;\mathrm{formation}$$

(3) $$3I^{-}+2h^{+}\to I_{3}^{-}\;\;\;\mathrm{Triiodide}\;\mathrm{formation}$$

(4) $$Pb^{2+}+2e^{-}\to Pb^{0}\;\;\;Pb^{0}\;\mathrm{formation}$$

(5) $$\begin{array}{c} \begin{array}{c} \mathrm{MAPb}{\left(I_{0.5+x}Br_{0.5-x}\right)}_{3}\overset{h\nu }{↔}\left(0.5-x\right)\mathrm{MAPbB}r_{3}+\left(0.5+x\right)\mathrm{MAI}\left(\mathrm{gas}\right)\\ \;\;+\left(0.5+x\right)Pb^{0}+\frac{0.5+x}{2}I_{2}\;\;\;\mathrm{Reconstruction} \end{array} \end{array}$$

Equation (1) corresponds to the phase segregation of MAPb(I_0.5_B_0.5_)_3_ under blue‐light irradiation. Equations (2) and (3) correspond to the oxidization of iodide and the formation of molecular iodine and triiodide. Since I_2_ is volatile, it tends to migrate to the surface and then escape from the perovskite thin films, making this oxidation process a chain reaction. The expulsion of I^−^ from the lattice results in the decomposition of the I‐rich domains and induces the reconstruction of Br‐rich domains, forming MAPbBr_3_ phases (Equation (5) and Figure 5E). Meanwhile, under blue‐light irradiation, the escape of volatile MAI, and photolysis of PbI_2_ may also simultaneously occur.^[^ ^48^, ^53^ ^]^

With the blue‐light irradiated on the thin films, only one PL peak can be observed at 1.70 eV indicating efficient energy transfer processes in the mixed‐halide perovskite thin films. To confirm this, we fabricate a structure as shown in Figure 5F. The MAPbBr_3_ thin film is prepared by solution process, and the MAPbI_3_ part is deposited on the MAPbBr_3_ thin film by a vapor‐assisted process.^[^ ^54^ ^]^ The PL spectra of this structure are shown in Figure 5G,H. Intriguingly, only one peak can be found in the PL measurements both from the front and back sides, which is consistent with the PL emission of MAPbI_3_. Moreover, we also put the thin films under blue‐light irradiation. The PL spectra slightly shift to the longer wavelength both from the front and back sides. Considering the band alignment (Figure 5F), this result shows that the energy transfer process is efficient in this structure and only the lowest energy PL emission can be observed in mixed‐halide perovskites. Moreover, it is found that the evaporated MAPbI_3_ thin film is more stable than the solution process one. This structure can stay more than half an hour without decomposition under the same blue‐light irradiation. Furthermore, we also prepare MAPb(I_1−_ *_x_* Br*_x_*)_3_ thin films with a vapor‐assisted process, and the PL evolution is presented in the Supporting Information under continuous blue‐light irradiation (Figures S15 and S16, Supporting Information). It took almost 2 h for the emission to evolve from red to green. Considering the evaporated thin films are more compact than the solution‐processed ones, we speculate that compact thin films with fewer defects and grain boundaries are more stable than the poriferous thin films which are apt to phase segregation and decomposition. Furthermore, the difference could be relevant to the residual solvent such as dimethyl sulfoxide (DMSO) in the solution‐processed films. It was reported that DMSO forms a complex on the surface of perovskite grains, and is regarded as the weak point in the films.^[^ ^55^ ^]^

Carrier recombination has several relaxation channels, such as band‐to‐band recombination, trap‐assisted recombination, Auger recombination, and surface recombination. These channels can be classified into radiative and nonradiative. In the former, part of the energy is released by photons, whereas the latter converts the excess energy into heat. One key challenge is the need to suppress nonradiative recombination for perovskite optoelectronic devices in polycrystalline thin films. For instance, nonradiative recombination introduces a loss to the open‐circuit voltage of a solar cell device which will directly decrease the device PCE. Here, we show a radiative increase state during the decomposition process in mixed‐halide perovskites, which can potentially improve the related device performance. The photobrightening effect has been shown in a number of reports.^[^ ^48^, ^56^, ^57^ ^]^ This effect was considered to be induced by the migration/interdiffusion of different halides.^[^ ^58^, ^59^, ^60^ ^]^ As iodine has a lower ionic migration activation energy than bromine, its migration rates surpass bromine in the illuminated area.^[^ ^35^, ^51^ ^]^ Thus, it is possible that unreacted or free iodine goes out of and bromine comes into the illuminated area. Here, we propose the increased emission is due to the formation of type‐I band alignment between the highly segregated I‐rich and Br‐rich domains. This energy band alignment significantly suppresses the nonradiative recombination in I‐rich domains and grain boundaries, and correspondingly enhances the PL intensity. To confirm this mechanism, we conduct the same experiments on the MAPb(I_0.5_Br_0.5_)_3_ samples which have a 100 nm Polymethyl Methacrylate (PMMA) on top (Figure S17, Supporting Information). With the protection of PMMA, the atmosphere effect can be excluded. Interestingly, the photobrightening effect can still be observed in a few minutes, which can eliminate the impact of the atmosphere. Previously, the photobrightening phenomena are all observed in MAPbI_3_ or MAPbBr_3_. To our knowledge, this is the first time to observe the photobrightening process in the mixed‐halide thin films. The passivation of nonradiative decay channels can provide potential applications for mixed‐halide perovskites in tandem solar cells. However, the formation of I‐rich and Br‐rich domains causes another issue (i.e., phase reconstruction). This band alignment induces the charge injection into I‐rich domains leading to the irreversible MAPbBr_3_ reconstruction. We deduce that this may be the name of the game for the instability of mixed‐halide perovskites. Thus, how to eliminate the formation of I‐rich and Br‐rich domains has become the key to the practical utilization of mixed‐halide perovskites.

In summary, we identify and monitor the phase reconstruction processes of mixed‐halide perovskites under continuous blue‐light irradiation. Due to the charge injection from Br‐rich to I‐rich domains, the formation of molecular iodine is induced, leading to the reconstruction of MAPbBr_3_. Since I_2_ is volatile and tends to escape from the perovskite thin films, the reconstruction proceeds as a chain reaction. We propose that the reversible photoinduced phase segregation in mixed‐halide perovskites is inevitably accompanied by such irreversible phase reconstruction processes, which can be regarded as a universal intrinsic self‐evolution pathway. To achieve the long‐term stability of perovskite‐based electronics, it is vital to develop strategies to suppress the migration and oxidization of halide ions. This work provides significant information for the phase segregation and further reconstruction of perovskites and sheds light on understanding the property evolution.

## 3 Experimental Section

### Materials

Lead iodide (PbI_2_, 99.99%) was purchased from TCI. Lead bromide (PbBr_2_), methylammonium iodide (MAI), and methylammonium bromide (MABr) were purchased from Xi'an Polymer Light Technology Corp. *N*,*N*‐Dimethyl formamide (DMF, 99.8%), and DMSO (99.7%) were purchased from Acros. The fluorine doped tin oxide (FTO) glass substrates were purchased from Yingkou OPV Tech New Energy Co., Ltd. All the materials were used as received without further purification.

### Precursor Preparation

The perovskite precursors were prepared by dissolving PbI_2_, PbBr_2_, MAI, MABr in DMF/DMSO (volume ratio 4:1) co‐solvent, and stirring at 70 °C for 30 min. The ratio of (PbBr_2_+MABr)/(PbBr_2_+PbI_2_+MAI+MABr) was controlled to be 1/10, 2/10, 3.5/10, 5/10, 6.5/10, and 8/10. The concentration of Pb^2+^ was fixed at 0.3 m.

### Perovskite Film Preparation

The mixed‐halide perovskite films were prepared by spin‐coating the precursors on ITO or cover glass substrates. Since the perovskite precursors have a quite low concentration, it is unnecessary to conduct the antisolvent treatment to obtain smooth and compact perovskite thin films. The precursor was spun on the glass substrates at 5000 rpm (ramp 2000 rpm) for 30 s and then annealed at 100 °C for 5 min. Since MAPbBr_3_ has a much lower solubility than MAPbI_3_ in DMF/DMSO, the concentration of the perovskite precursors was fixed at 0.3 m. The thickness of the as‐deposited perovskite films was around 80 nm.

### MAPbI_3_/MAPbBr_3_ Bilayer Preparation

The MAPbBr_3_ film was deposited on the glass substrate by the solution process, and the MAPbI_3_ film was deposited on the top of MAPbI_3_ film by a vapor–solid reaction technique. The perovskite precursor of MAPbBr_3_ was spun on the glass substrate at 5000 rpm (ramp 2000 rpm) for 30 s and then annealed at 100 °C for 5 min. Then, 50 nm of PbI_2_ film was deposited on the as‐prepared MAPbBr_3_ film by thermal evaporation with a rate of 0.2 Å s^−1^. The samples of glass/MAPbBr_3_/PbI_2_ were transferred into a home‐built vacuum chamber. In the chamber, a glass substrate holding MAI powders was placed underneath the samples. The distance between the sample of glass/MAPbBr_3_/PbI_2_ and the MAI holder was fixed to be 2.5 cm. To form a MAI atmosphere in the chamber and trigger the reaction between PbI_2_ and MAI, the temperature in the chamber was increased to 160 ℃, and the pressure in the chamber was decreased to ≈100 Pa. After 30 min, the PbI_2_ was transformed into MAPbI_3_ completely, and the preparation of glass/MAPbBr_3_/MAPbI_3_ samples was completed. Notably, it is possible that anion exchange process occurs at the interface between MAPbI_3_ and MAPbBr_3_, which induces the formation of a mixed‐halide perovskite layer. The potential influence of this issue is currently under investigation.

### Characterization

PL measurements: Typically, the PL spectra were obtained using a Halogen lamp (U‐LH100‐3, Olympus) as excitation after passing through an ≈460–490 nm bandpass filter, and the excitation power under the 100X objective is estimated to be ≈1.04 W cm^−2^ that was measured by an optical powermeter. To observe the initial PL spectra and photoinduced evolution of PL spectra, a CW 532 nm laser through a 5X objective (0.15 NA) was used as the excitation, and the excitation power was tuned to be 680 and 68 mW cm^−2^. Notably, the PL spectra of MAPbBr_3_ were always collected using Halogen lamp as the excitation, since its emission is below 532 nm. To capture the PL imaging results, a mercury lamp (U‐LH100HG, Olympus) was used as the excitation after passing through an ≈460–490 nm band‐ass filter, and the excitation power under the 100X objective is estimated to be ≈13.0 W cm^–2^. The signal passed through a 520 nm long‐pass filter before collected by a CCD camera (Tucsen, TCH‐1.4CICE) or a spectrometer (Renishaw inVia). The XRD spectra were measured with an X'pert PRO X‐ray diffractometer by Cu K*α* radiation under conditions of 40 mA and 40 kV with a range from 5° to 55°, the scanning speed is 5° min^−1^. The SEM was obtained by a Nova NanoSEM 450 field emission SEM. The UV–visible absorption (UV–vis) spectrum was tested by a UV–vis–NIR spectrophotometer (SolidSpec‐3700). The XPS spectra were collected by X‐ray photoelectron spectrometer (Kratos AXIS Supra). The samples were cut into 5 × 5 mm^2^ sizes, and stick them on the sample stage with tape. The voltage when collecting the full spectra is 15 kV, and the current is 5 mA. 15 kV and 10 mA when collecting the spectra for each element. ToF‐SIMS measurements were based on the focused ion beam ToF‐SIMS spectrometer (GAIA3 GMU Model 2016, Czech).

## Conflict of Interest

The authors declare no conflict of interest.

## Author Contributions

Z.L. and X.Z. contributed equally to this work. Y.R. and T.W. conceived the idea and designed the experiments; Z.L. conducted the PL imaging measurements and processed the experiment data; X.Z., X.X., and Q.H. contributed to the sample preparation; Y.R., Y.A., and Q.A. conducted XRD, XPS, and SEM measurements. Y.W. conducted TOF‐SIMs measurements. H.X. provided funding and equipment for this project. All authors were involved in the discussion of the results. Y.R. and T.W. wrote the manuscript, and X.L., R.C. helped with revising.

## Supporting information

Supporting Information

Supplemental Video 1

## Data Availability

The data that support the findings of this study are available from the corresponding author upon reasonable request.
