# Supplementary material for: Beyond the Phase Segregation: Probing the Irreversible Phase Reconstruction of Mixed‐Halide Perovskites
Source: Adv Sci (Weinh). 2021 Dec 19;9(5):2103948. doi: 10.1002/advs.202103948 (PMC8844510; doi:10.1002/advs.202103948)
Supplement: Supplementary file 1 — Supporting Information [file ADVS-9-2103948-s002.pdf]

ADVANCED SCIENCE
Open Access

Supporting Information

for *Adv. Sci.*, DOI: 10.1002/advs.202103948

Beyond the phase segregation: probing the irreversible phase reconstruction of mixed-halide perovskites

Zhe Li[1,†], Xin Zheng[2,†], Xuan Xiao[2], Yongkang An[3], Yanbo Wang[4], Qingyi Huang[2], Xiong Li[2], Rongrong Cheacharoen[5], Yue Hu[2], Qinyou An[3], Yaoguang Rong[2,*], Ti Wang[1,*], Hongxing Xu[1,*]

[1]School of Physics and Technology and Key Laboratory of Artificial Micro- and Nanostructures of Ministry of Education, Wuhan University, Wuhan 430072, China.
[2]Wuhan National Laboratory for Optoelectronics, Huazhong University of Science and Technology, Wuhan 430074, China.
[3]State Key Laboratory of Advanced Technology for Materials Synthesis and Processing, Wuhan University of Technology, Wuhan 430070, Hubei, China
[4]State Key Laboratory of Metal Matrix Composites, Shanghai Jiao Tong University, Shanghai 200240, China
[5]Metallurgy and Materials Science Research Institute, Chulalongkorn University, Bangkok, 10330, Thailand
[†]These authors contributed equally

*Correspondence:
ygrong@hust.edu.cn
wangti@whu.edu.cn

hxxu@whu.edu.cn

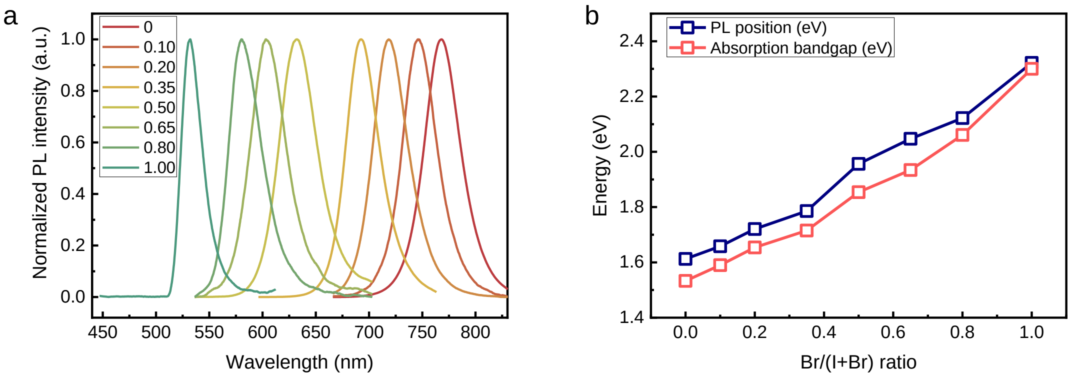

Figure S1. (a) The initial PL spectra of $MAPb(I_{1-x}Br_x)_3$ films with different x values. In order to collect the spectra before noticeable photo-induced spectra changes have occurred, the excitation and integration time was shortened to 100 ms. It is noted that the noise level in the original data is high due to the much shorter integration time, and the spectra curves are smoothed by Savitzky-Golay method. (b) the comparison of the initial PL positions and bandgaps of $MAPb(I_{1-x}Br_x)_3$.

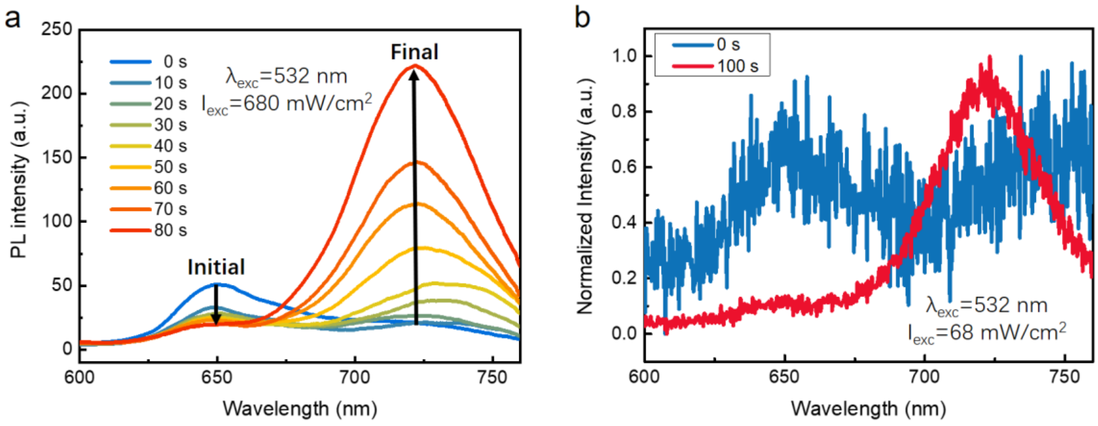

Figure S2. (a) Evolution of the PL spectra of $MAPb(I_{0.5}Br_{0.5})_3$ films under a low intensity excitation (680 mW cm^{-2}) with an integration time of 10 seconds. (b) Evolution of the PL spectra of $MAPb(I_{0.5}Br_{0.5})_3$ under a lower intensity excitation (68 mW cm^{-2}) with an integration time of 10 seconds. The samples are excited with a CW 532 nm laser through a 5X objective (0.15 NA).

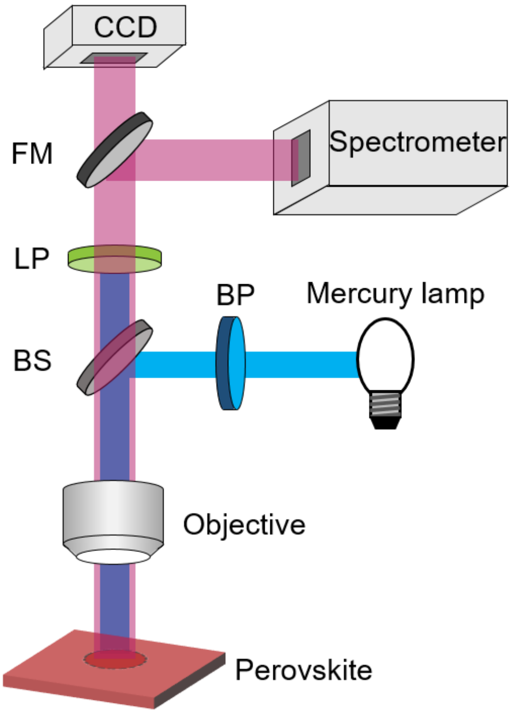

Figure S3. Schematic of the PL imaging microscopy experiment setup. BP: bandpass filter; BS: beam splitter; LP: long pass filter; FM: flip mirror; CCD: charge coupled device.

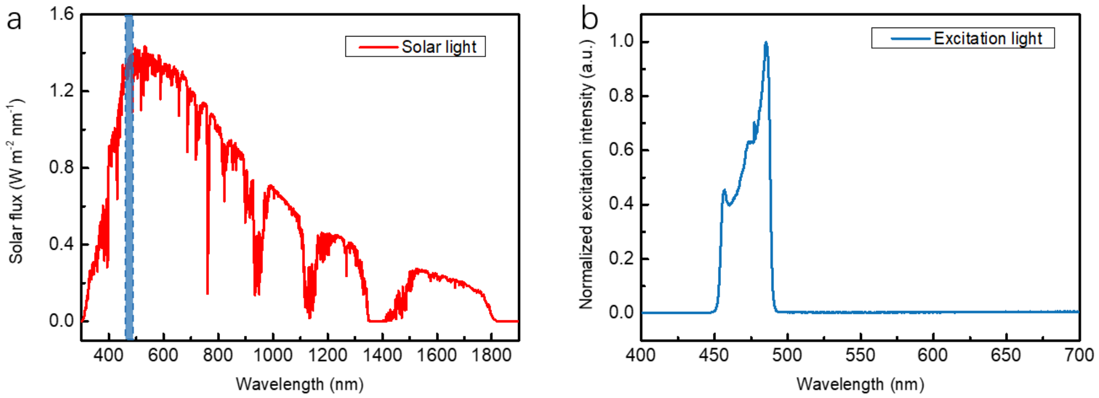

Figure S4. The spectrum of solar light (a) and irradiation light (b) used in the experiment. The blue area in (a) indicates the wavelength range of (b).

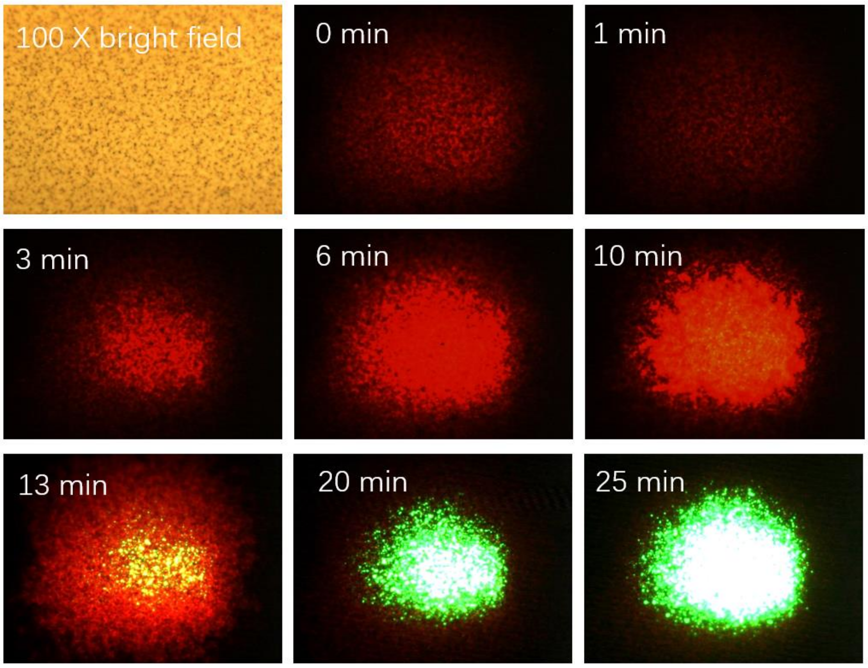

Figure S5. PL images of the $MAPb(I_{0.35}Br_{0.65})_3$ film under continuous irradiation. A similar reconstruction process is observed.

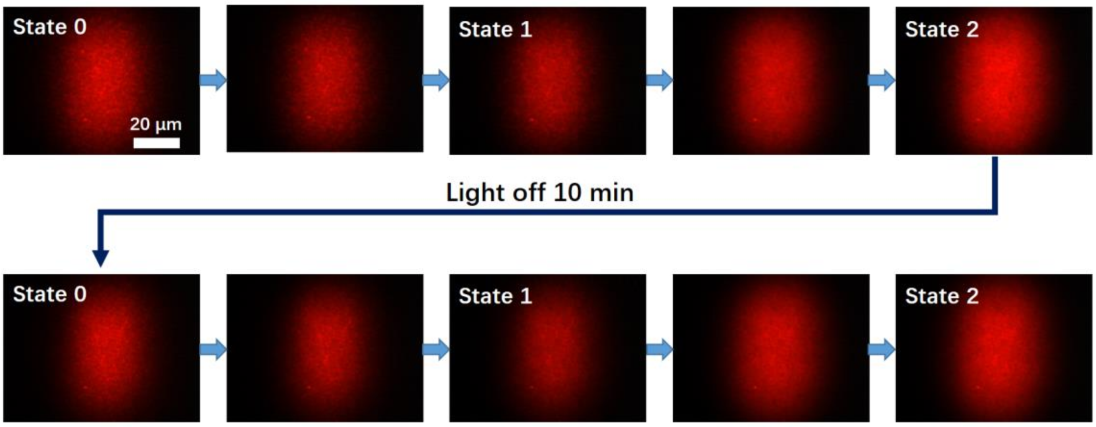

Figure S6. PL images of the $MAPb(I_{0.5}Br_{0.5})_3$ film under continuous irradiation. The whole process is still reversible until State-2.

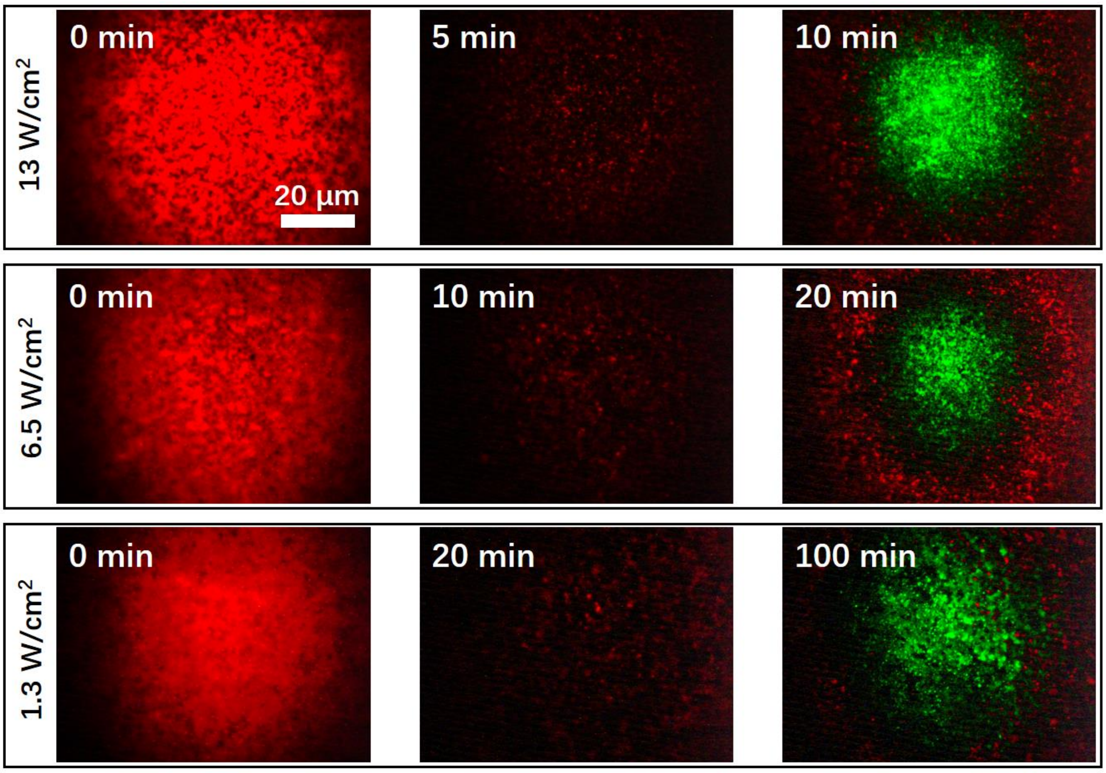

Figure S7. Power and time dependence PL images of $MAPb(I_{0.5}Br_{0.5})_3$ film under continuous irradiation. Compare with the 13 W/cm^2 case, the sample spend 2 and 10 times time to reach the similar state under 6.5 W/cm^2 and 1.3 W/cm^2 power, indicating the phase reconstruction is proportional to the light flux.

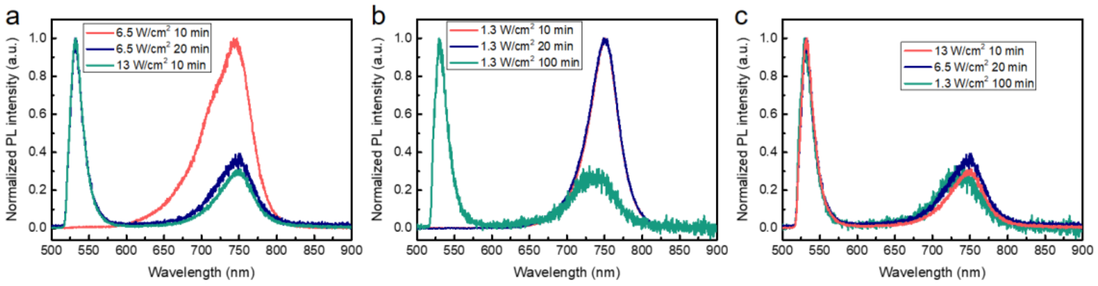

Figure S8. Power and time dependence of the PL spectra of $MAPb(I_{0.5}Br_{0.5})_3$ films under continuous irradiation.

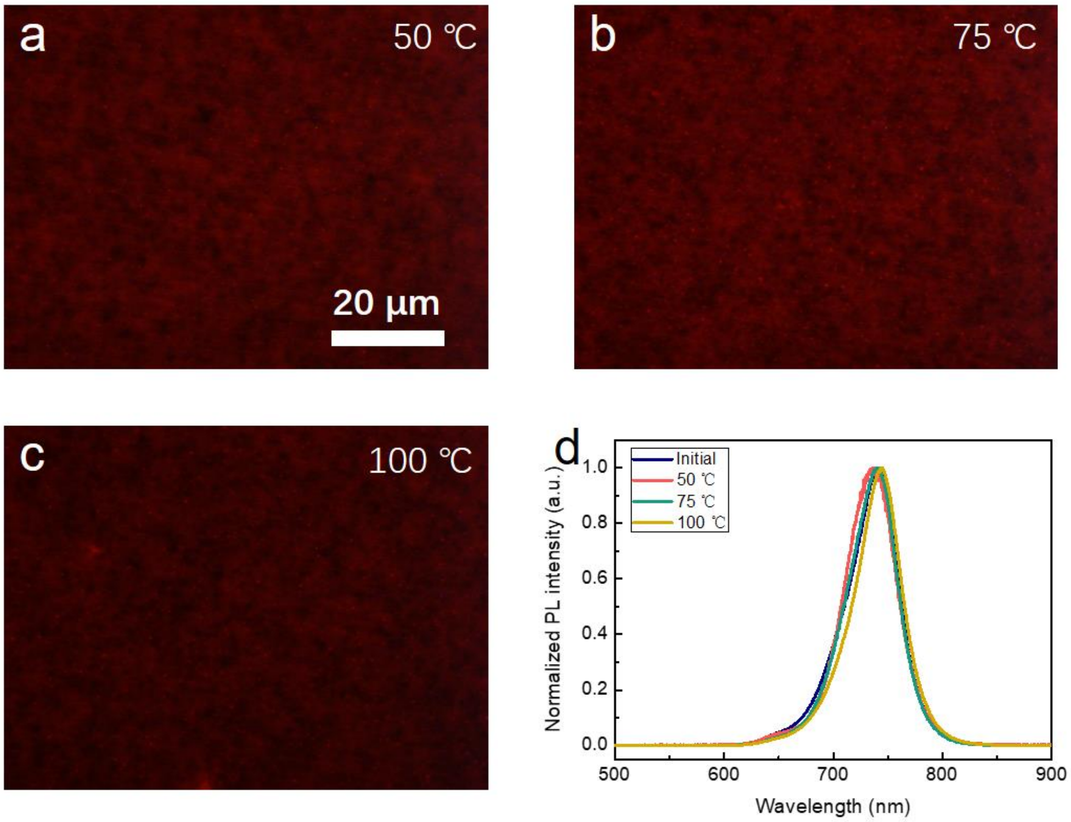

Figure S9. PL images and spectrum (d) of the $MAPb(I_{0.5}Br_{0.5})_3$ thin film after 10 min heating under (a) 50 ℃, (b) 75 ℃ and (c) 100 ℃.

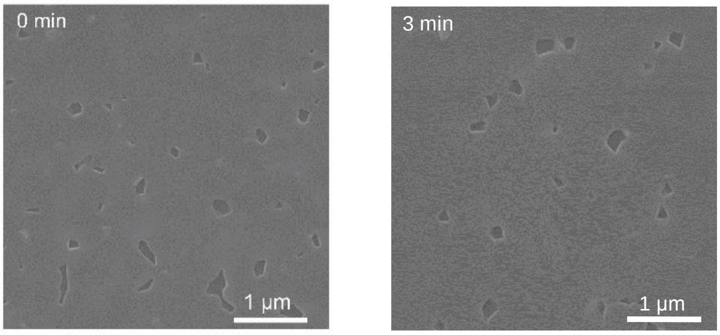

Figure S10. The surface SEM image of $MAPb(I_{0.5}Br_{0.5})_3$ thin films irradiated by blue excitation light for 0 and 3 min.

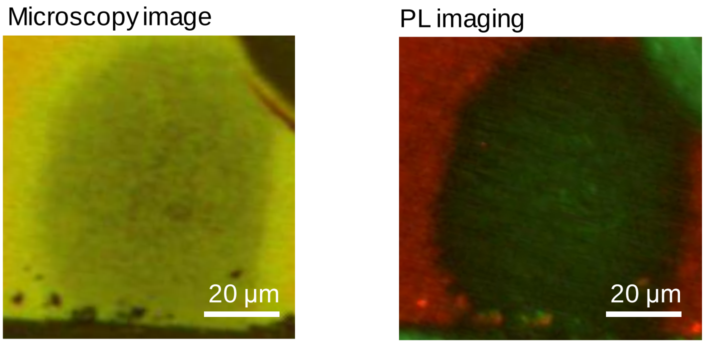

Figure S11. The microscopy and PL imaging of the irradiated area on the $MAPb(I_{0.5}Br_{0.5})_3$ film.

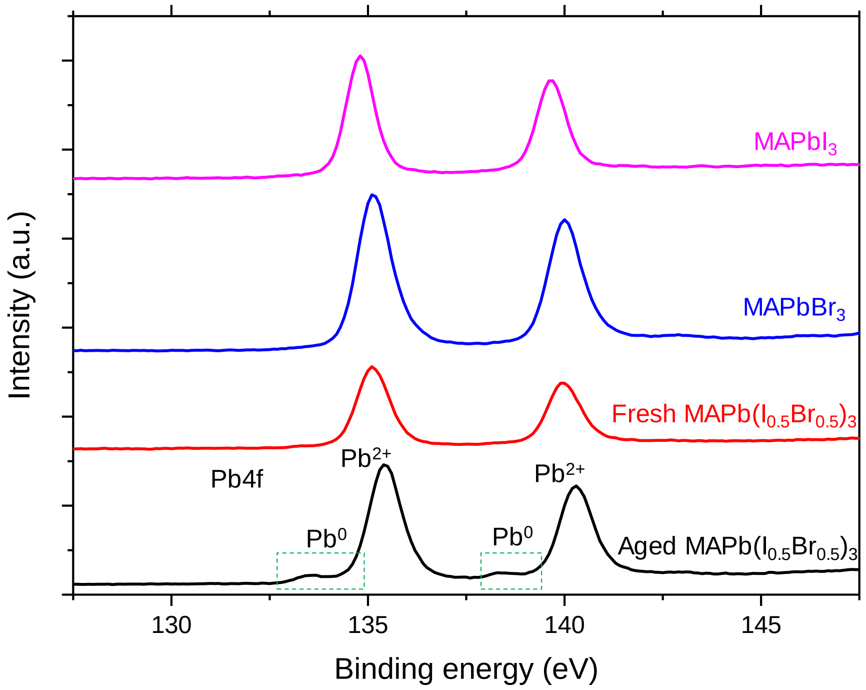

Figure S12. The XPS spectra of $MAPbI_3$, $MAPbBr_3$, fresh $MAPb(I_{0.5}Br_{0.5})_3$ and aged $MAPb(I_{0.5}Br_{0.5})_3$ films.

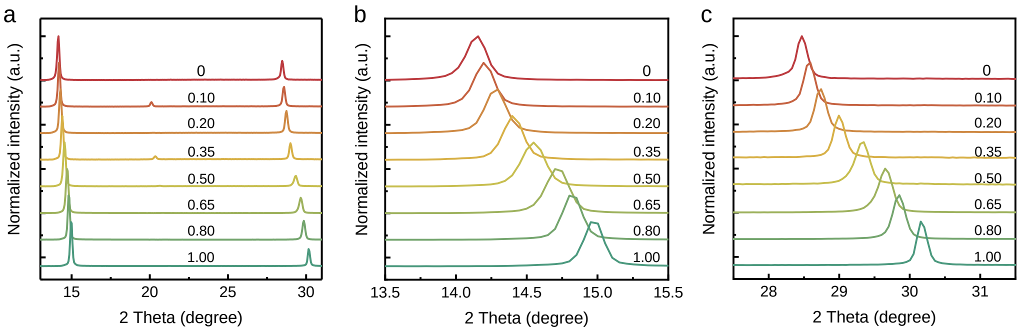

Figure S13. The XRD patterns of fresh $MAPb(I_{1-x}Br_x)_3$ films with different x values.

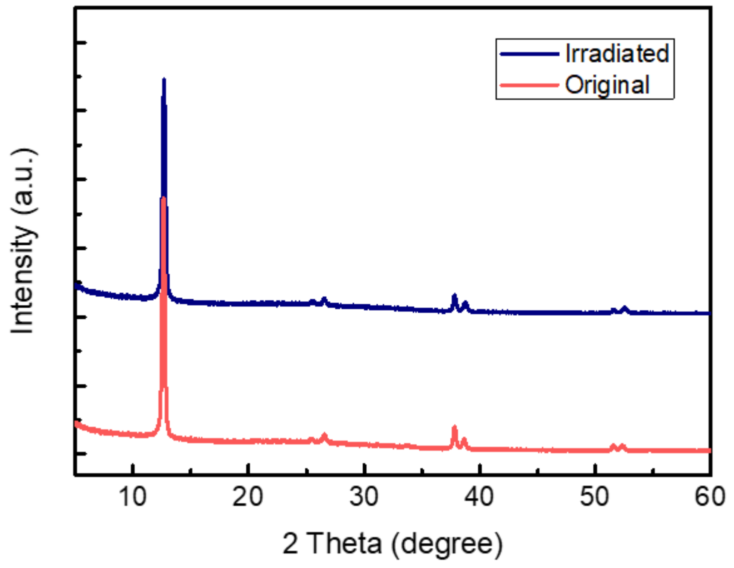

Figure S14. XRD patterns of fresh PbI_2 film and aged PbI_2 film (Mercury lamp, 30 min).

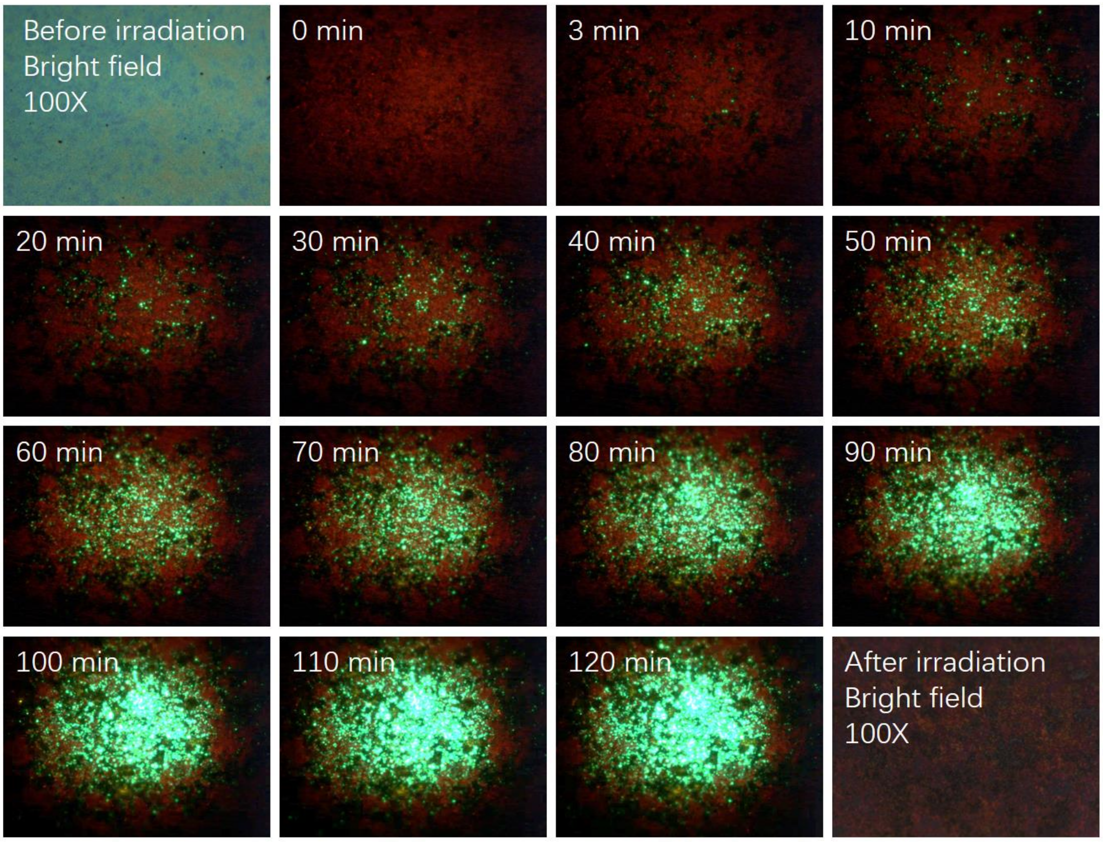

Figure S15. PL images of the $MAPb(I_{1-x}Br_x)_3$ thin film deposited by vapor-assisted process under continuous irradiation.

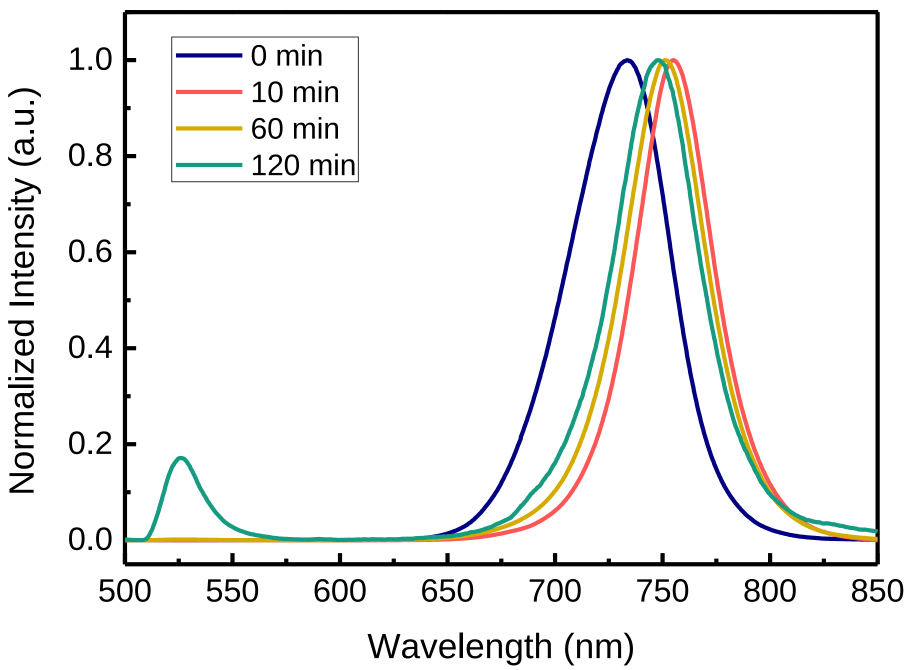

Figure S16. The PL spectra of the $MAPb(I_{1-x}Br_x)_3$ thin film deposited by vapor-assisted process under continuous irradiation.

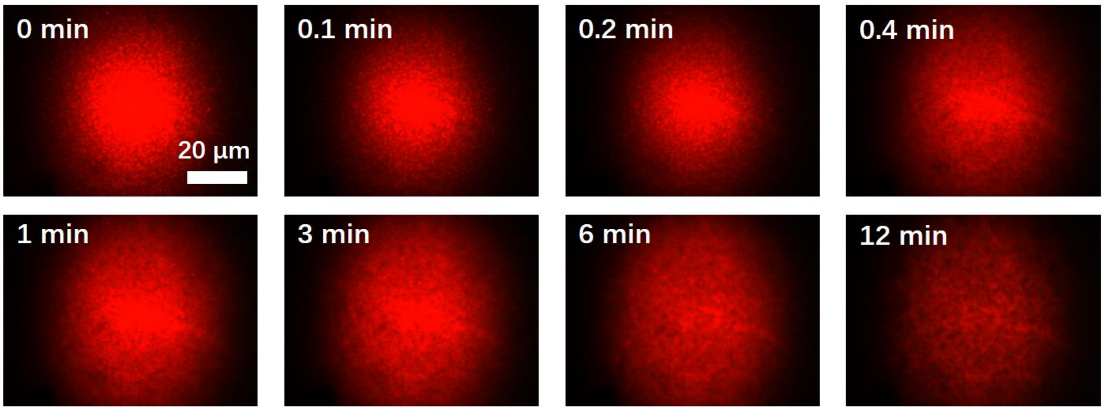

Figure S17. PL images of the PMMA coated $MAPb(I_{0.5}Br_{0.5})_3$ film under continuous irradiation. No reconstruction is observed as I_2 escape is prevented by the PMMA film.
